# Advanced Diagnostic Technologies and Molecular Biomarkers in Periodontitis: Systemic Health Implications and Translational Perspectives

**DOI:** 10.3390/jcm15031142

**Published:** 2026-02-02

**Authors:** Sebastian Biesiadecki, Monika Janeczko, Joanna Kozak, Magdalena Homaj-Siudak, Lukasz Szarpak, Mansur Rahnama-Hezavah

**Affiliations:** 1 Department of Clinical Research and Development, LUXMED Group, 02-678 Warsaw, Poland; sebastianbiesiadecki123@gmail.com (S.B.); magdalena.homaj@gmail.com (M.H.-S.); 2 Institute of Biological Sciences, The John Paul II Catholic University of Lublin, 20-708 Lublin, Poland; monika.janeczko@kul.pl; 3 Institute of Medical Science, The John Paul II Catholic University of Lublin, 20-708 Lublin, Poland; joanna.kozak@kul.pl; 4 Department of Dental Surgery, Medical University of Lublin, 20-093 Lublin, Poland

**Keywords:** periodontitis, salivary biomarkers, gingival crevicular fluid, point-of-care diagnostics, biosensors, microRNA, cell-free DNA, extracellular vesicles, oral microbiome, artificial intelligence

## Abstract

**Background/Objectives:** Periodontitis is a chronic inflammatory disease with marked inter-individual heterogeneity and well-established links to cardiometabolic and other systemic conditions. Conventional clinical diagnostics remain indispensable. However, they provide limited real-time insight into molecular activity and host-response biology. This review aimed to synthesize recent advances in point-of-care diagnostics and emerging molecular biomarkers relevant to periodontal disease and its systemic associations. **Methods:** We performed a state-of-the-art narrative review of literature published between 2018 and 2026. The focus was on point-of-care biosensing technologies and molecular biomarkers assessed in oral and related biological matrices. These included saliva, gingival crevicular fluid, blood, and dental plaque. Evidence was prioritized based on analytical performance, clinical validity, and translational readiness. **Results:** Substantial progress has been made in multiplex optical and electrochemical point-of-care platforms. These include microfluidic systems and early intraoral wearable sensors. Such technologies enable quantification of host-response proteins, including MMP-8, cytokines, and chemokines. In parallel, omics-derived biomarkers are emerging as clinically informative adjuncts for diagnosis and monitoring. MicroRNAs, cell-free DNA, extracellular vesicle–derived signals, proteomic profiles, and microbiome classifiers demonstrate promising discrimination. They also provide mechanistic links to systemic inflammation. Clinical translation remains limited by study heterogeneity, spectrum bias, and insufficient external validation. **Conclusions:** Near-term clinical value lies in adjunctive risk stratification and longitudinal disease monitoring. Replacement of conventional periodontal examination is not currently justified. Meaningful clinical and public-health impact will require standardized disease definitions. Harmonized sampling and reporting protocols are essential. Multicenter validation across comorbidity strata is needed. Regulatory-grade evidence must be generated for in vitro diagnostics and artificial intelligence software classified as medical devices.

## 1. Introduction

Periodontitis is a chronic, multifactorial inflammatory disease driven by oral microbiome dysbiosis and an exaggerated host immune response. A key effector arm of periodontal tissue injury is neutrophil activation with release of proteolytic enzymes, including collagenases, which directly link inflammatory burden to connective tissue breakdown. In parallel, macrophage inflammasome activation contributes to IL-1β maturation and can be accompanied by pyroptotic cell death, amplifying local inflammation and tissue damage. These pathways provide a mechanistic rationale for host-response biomarkers such as aMMP-8 and IL-1β as clinically relevant readouts of ongoing destructive inflammation. The 2018 classification emphasizes disease staging and grading, enabling precise assessment of severity, complexity, and risk of progression [1]. Despite this framework, routine dental diagnostics still rely mainly on macroscopic indicators of past tissue damage. These include probing depth, clinical attachment loss, radiographic bone loss, and general activity markers such as bleeding on probing [2]. Such measures are essential for diagnosis and treatment planning. However, these measures provide limited insight into short-term inflammatory dynamics and offer little ability to distinguish biologically distinct disease endotypes that may respond differently to therapy [2,3]. The increasing interest in scalable screening and monitoring tools is therefore driven by well-established associations between periodontitis and systemic conditions, including cardiometabolic disease, diabetes, adverse pregnancy outcomes, autoimmune disorders, and respiratory infections [4,5,6].

Early detection, risk stratification, and longitudinal monitoring are clinically relevant in patients with multimorbidity [4,7]. Recent advances follow two principal directions. First, advanced diagnostic technologies have emerged, especially point-of-care biosensors and integrated platforms detecting host-response markers. Salivary or gingival crevicular fluid biomarkers, such as IL-1β, MMP-8 (including its active form, aMMP-8), and calprotectin, enable rapid, noninvasive, multimarker assessment of inflammatory activity and risk stratification [1,2,7,8,9]. Beyond inflammatory mediators, markers of alveolar bone resorption are clinically relevant, as bone loss ultimately drives long-term morbidity. The RANKL/OPG axis governs osteoclast activation and links immune signalling to hard-tissue destruction; accordingly, elevated RANKL and/or an increased RANKL/OPG ratio in saliva or gingival crevicular fluid has been associated with active periodontal breakdown and may complement inflammatory panels when estimating progression risk. Second, novel molecular biomarkers are under investigation. These include microRNAs, cell-free DNA, extracellular vesicles, proteomic profiles, and microbiome-based classifiers [1,2,7,8,9,10,11,12,13,14]. Such approaches allow detection of subclinical changes, prediction of progression, and support therapy personalization. Importantly, several longitudinal observations suggest that molecular signals may precede clinically detectable attachment loss. In periodontally healthy individuals, elevated neutrophil-driven proteolytic activity (e.g., increased active MMP-8) and the carriage or enrichment of selected periopathogens have been associated with a higher likelihood of subsequent periodontal breakdown. These ‘pre-clinical’ signals are clinically attractive because they shift the diagnostic focus from documenting past tissue loss to identifying patients at risk before irreversible damage occurs, thereby supporting more targeted prevention and earlier intervention. The translational readiness of these solutions continues to increase. Their integration into patient-centred care models supports more precise clinical decision-making. This is particularly relevant in the context of interdisciplinary care, where periodontal status must often be considered alongside systemic disease management.

The aim of this review is to provide clinicians with a practical perspective on which emerging diagnostic tools may already support patient care in the near term, and which should, for now, remain confined to research or specialist settings.

**Figure 1:**
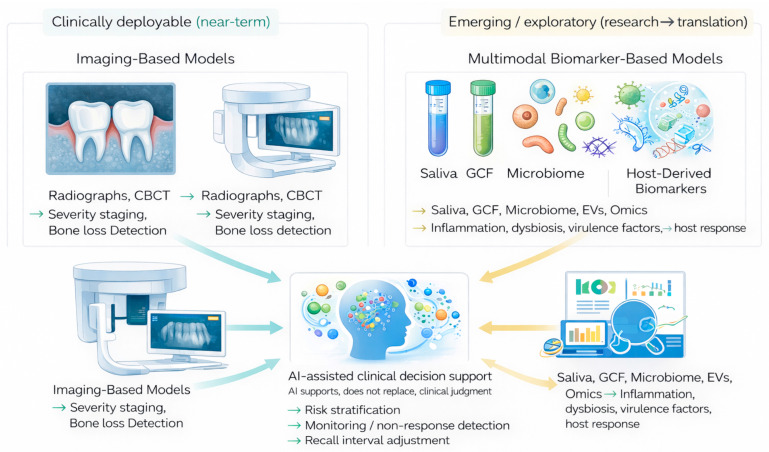
**Translational framework of advanced periodontal diagnostics.** Clinically deployable imaging-based models and emerging multimodal biomarker approaches converge into AI-assisted clinical decision support. Imaging-based models are currently more mature, while multimodal biomarker-based models remain largely exploratory. AI systems support—rather than replace—clinical judgement and may inform risk stratification, monitoring, and personalized recall strategies. Created in BioRender. Szarpak, L. (2026) https://BioRender.com/fv469x8.

To provide a conceptual framework for the developments discussed below, Figure 1 summarizes the translational pathway of advanced periodontal diagnostics, highlighting how conventional clinical measures, host-response biomarkers, molecular profiling, and AI-supported decision tools may converge to support risk stratification, monitoring, and personalized clinical management.

## 2. Materials and Methods

### 2.1. Review Design

This manuscript is a state-of-the-art narrative review integrating recent technological and biological advances relevant to periodontal diagnostics and systemic health. The objective was translational synthesis across heterogeneous domains (point-of-care biosensors, molecular biomarkers, microbiome/functional profiling, and AI-supported integration), rather than an exhaustive systematic review of a single intervention or outcome. Accordingly, no protocol was registered and no meta-analysis was performed. Given the breadth of technologies, study designs, and endpoints, the review was not designed as a PRISMA-guided systematic review; instead, we provide a transparent description of information sources, search strategy, evidence prioritization, and critical appraisal. Full database-specific search strings are reported in Supplementary Table S1.

### 2.2. Information Sources and Search Strategy

Relevant literature was identified through structured searches of PubMed/MEDLINE and Scopus, supported by backward and forward citation tracking of key primary studies, consensus statements, and high-quality reviews. The primary coverage window was 1 January 2018 to 10 January 2026. Searches were limited to English-language publications. Earlier seminal studies were included selectively when required to provide biological context or to anchor core diagnostic concepts.

Search terms combined “periodontitis” with domain-specific concepts related to oral fluid diagnostics and emerging technologies, including: point-of-care testing, biosensors, multiplex platforms, saliva and gingival crevicular fluid biomarkers, extracellular vesicles/exosomes, miRNA, cell-free DNA, DNA methylation, proteomics, microbiome/metagenomics/metatranscriptomics, prediction/progression, and artificial intelligence/machine learning. Complete search strings for each database are provided in Supplementary Table S1.

### 2.3. Evidence Prioritization and Synthesis Approach

Evidence was screened pragmatically for clinical relevance and translational maturity. Priority was given to human studies evaluating biomarkers in saliva, gingival crevicular fluid, blood, and dental plaque, and to studies reporting clinically meaningful outcomes (disease activity, progression, risk stratification, or response/non-response to therapy). Diagnostic accuracy studies were prioritized when they used a clearly defined reference standard and provided clinically interpretable performance measures. Systematic reviews, consensus statements, and guideline documents were used to contextualize the evidence where clinically relevant.

AI/machine-learning studies were included only when they applied clearly defined clinical labels and reported appropriate validation (e.g., separation of training and testing, cross-validation with clear methodology, and/or independent or held-out test datasets), with sufficient reporting to judge generalisability. Findings were synthesized narratively and organized by diagnostic platform and biomarker class. Biomarkers were highlighted based on frequency of reporting, replication across independent cohorts (or pooled evidence where available), and clinical interpretability of performance metrics in human samples. Findings limited to small single-centre case–control studies were treated as exploratory. Translational readiness was discussed across analytical validity, clinical validity, clinical utility, and implementation/regulatory feasibility, and limitations were stated explicitly where evidence remains early-stage.

To avoid overinterpretation of diagnostic performance metrics, we classified evidence maturity for each domain as: Exploratory (single-centre and/or case–control, limited sample, no external validation), Validated (independent cohort and/or external validation with consistent clinical labels), or Near-clinical (prospective/longitudinal evaluation and/or implementation-ready workflow with demonstrated incremental value over standard clinical assessment). When performance values (AUC, sensitivity/specificity) are cited, we specify whether they come from single studies or pooled/meta-analytic estimates, and we highlight relevant heterogeneity or methodological constraints. Practical inclusion and exclusion criteria (sample types and eligible study designs) are summarized in Supplementary Table S2.

### 2.4. Methodological Considerations

Given the narrative scope and heterogeneity of study designs, we did not apply a single formal risk-of-bias tool across all included studies. Instead, we used a clinically oriented critical appraisal to weight conclusions toward evidence with higher translational credibility. Greater emphasis was placed on prospective/longitudinal cohorts and studies with clinically meaningful endpoints (progression, response/non-response), clear reference standards, transparent pre-analytics and assay procedures, and appropriate validation (independent or held-out test datasets rather than internal cross-validation alone).

When interpreting diagnostic accuracy and biomarker data, we explicitly considered the most common threats to clinical translation in periodontology: spectrum bias typical of case–control designs, small and highly selected samples, inconsistent case definitions and reference standards, non-standardized sampling and analytical workflows, and limited external validation across diverse patient populations. Where evidence was discovery-stage or derived from small case–control datasets, it was framed as hypothesis-generating, and the text was tempered to avoid implying routine clinical readiness without independent validation and demonstrated incremental value over staging and grading. To prevent overinterpretation of diagnostic performance values reported across heterogeneous study designs, we summarize the principal diagnostic domains, the source of key performance claims (single study vs. pooled/meta-analytic), and overall evidence maturity in Supplementary Table S3. Accordingly, when reporting AUC, sensitivity, or specificity, we indicate the underlying study design and validation level and caution that estimates are setting-dependent and vulnerable to spectrum bias.

## 3. Results

### 3.1. Multiparametric Point-of-Care Diagnostic Platforms

Recent years have seen substantial progress in portable, low-cost biosensors enabling direct quantification of inflammatory mediators in saliva or gingival crevicular fluid (GCF) [8,15,16]. As outlined in the translational framework (Figure 1), host-response point-of-care platforms are currently the most clinically mature molecular tools available. From a practical standpoint, they are immediately relevant for adjunctive screening, risk stratification, and follow-up rather than for stand-alone diagnosis. Most of these systems focus on multiplex detection of host-response proteins, most commonly MMP-8 alongside selected interleukins and chemokines. The underlying sensing is usually optical or electrochemical, and in many cases the assays are coupled to microfluidic cartridges and simple, smartphone-based readouts, which makes their use feasible in routine clinical workflows [1,8,15].

#### 3.1.1. Optical Biosensors and Plasmonic Platforms

**Figure 2:**
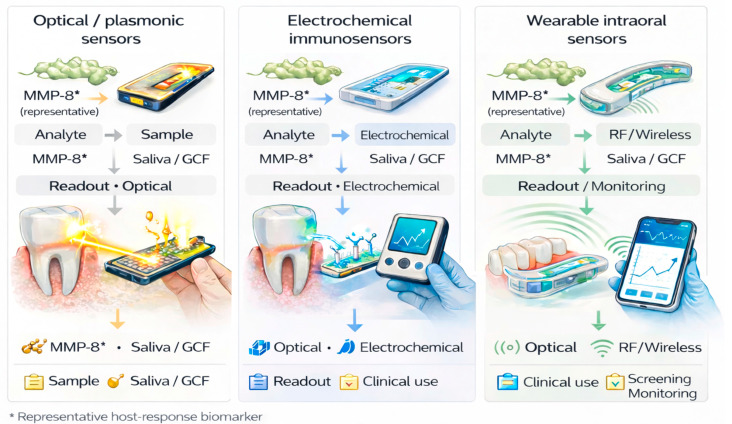
**Point-of-care diagnostic platforms for periodontitis.** Overview of optical, electrochemical, and wearable point-of-care technologies used for detection of host-response biomarkers in saliva or gingival crevicular fluid and their intended clinical applications. Created in BioRender. Szarpak, L. (2026) https://BioRender.com/fv469x8.

Plasmonic and fibre-optic platforms have been adapted for periodontal biomarker detection, achieving femtomolar limits of detection and clear multiplexing potential [16]. As illustrated in Figure 2, optical and plasmonic platforms allow for very sensitive detection of host-response biomarkers in saliva or gingival crevicular fluid. One illustrative example is the fibre-optic plasmonic point-of-care assay developed by Annunziata and colleagues for salivary macrophage inflammatory protein-1 alpha (MIP-1α). In analytical testing, the system achieved femtomolar sensitivity and was able to reliably distinguish patients with periodontitis from those with periodontal health [16]. A subsequent clinical validation study employed a three-arm surface plasmon resonance platform on plastic optical fibres (SPR-POF). This platform showed strong agreement with reference laboratory immunoassays, supporting near-term translational feasibility [16]. For MMP-8 detection, the SPR-POF system achieved a limit of detection of 40 pM (1.76 ng/mL) in buffer. In saliva, the detection limit was 225 pM (9.9 ng/mL). High analytical selectivity was maintained in the presence of interfering analytes [16].

#### 3.1.2. Electrochemical Assays and Disposable Immunosensors

Electrochemical approaches offer scalable manufacturing and low per-test costs through screen-printed electrodes and disposable cartridges [8,17]. A representative voltammetric immunosensor for salivary MMP-8 achieved nanogram-per-millilitre detection ranges. The device used graphene-based screen-printed electrodes modified with gold nanostructures. This design illustrates a pragmatic route toward chairside protein quantification [8]. An integrated dual-channel electrochemical immunosensor allowed for simultaneous measurement of IL-1β and MMP-8 in saliva. From a practical perspective, the platform showed a wide working range and high analytical sensitivity across clinically relevant concentrations. Across clinically relevant concentration ranges, the system showed stable performance for both IL-1β and MMP-8, with close agreement to reference methods [8]. Importantly, measuring the two markers together added practical value, as combined readouts were better able to distinguish between different stages of periodontal disease than either marker alone [8]. A critical translational requirement remains the definition of clinically meaningful thresholds. Equally important is performance stability across variable saliva matrices, sampling conditions, and comorbidity profiles [15,18,19].

A meta-analysis of diagnostic accuracy studies showed comparable AUC values for MMP-8 in saliva and GCF. Reported AUC values ranged from 0.70 to 0.90. Sensitivity ranged from 0.49 to 0.84, while specificity ranged from 0.62 to 0.79 (meta-analysis of diagnostic-accuracy studies; heterogeneity across matrices/thresholds and case definitions) [18,19,20]. A surface acoustic wave–based MMP-8 biosensor achieved an AUC of 0.81 and diagnostic accuracy of 74.2%. This performance differentiated periodontitis and gingivitis from periodontal health (single biosensor study; platform-specific performance; cohort/setting-specific—interpret with spectrum bias in mind) [15]. For periodontitis versus health and gingivitis, the AUC increased to 0.86, with accuracy of 82.8% (single biosensor study; periodontitis vs. health + gingivitis; platform-specific—external validation not stated) [15]. Active MMP-8 (aMMP-8), using a 20 ng/mL threshold, has been reported as a practical discriminator between periodontal health and disease in clinical cohorts [21].

#### 3.1.3. Wearable Devices and Continuous Intraoral Monitoring

Intraoral wearable sensors, such as tooth-mounted devices, represent an emerging strategy for continuous or repeated inflammatory monitoring [22,23]. A mouthguard-based sensor using a molecularly imprinted polymer combined with electrochemical impedance spectroscopy was evaluated. When coupled with deep learning, the system demonstrated feasibility for monitoring salivary MMP-8 [22]. A radiofrequency-based hydrogel sensor targeting salivary hydrogen sulphide (H_2_S) has also been reported. H_2_S is produced by periodontal pathogenic bacteria. The device enabled wireless signal transmission to a mobile terminal. It also allowed simultaneous release of the antibacterial agent chlorhexidine [23]. Despite their promise, intraoral wearable sensors still face several practical limitations. In practice, the limitations are straightforward—signal stability, the harsh oral environment, and patient compliance all remain unresolved issues. Equally important is the challenge of interpreting continuously generated signals in a way that can be meaningfully aligned with established clinical reference standards [22,24,25].

Experience to date suggests that the main barriers to wider implementation are not limited to technology alone. Available studies remain small, and many biochemical sensors are still at an early stage of development. In addition, the lack of consistent evaluation frameworks makes comparison across studies difficult. Moving forward, progress will depend less on proof-of-concept demonstrations and more on robust clinical validation, standardized performance metrics, and incorporation of real-world data into assessment pathways [24,25].

### 3.2. Emerging Molecular Biomarker Classes

**Figure 3:**
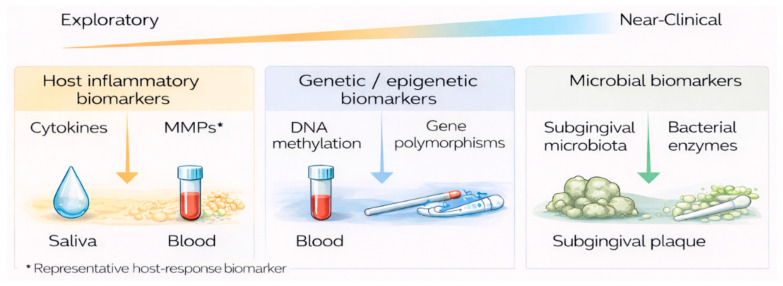
Emerging molecular biomarkers and their clinical readiness. Schematic overview of major classes of emerging molecular biomarkers for periodontitis, positioned along a translational readiness continuum from exploratory to near-clinical application. The figure highlights primary biological sample sources and key barriers to clinical implementation, including heterogeneity, limited standardization, and insufficient external validation. Created in BioRender. Szarpak, L. (2026) https://BioRender.com/fv469x8.

Figure 3 provides an overview of how emerging molecular biomarker classes currently fit into clinical practice rather than a simple catalogue of candidate markers. In the context of the broader translational framework shown in Figure 1, these biomarkers occupy an intermediate position: biologically informative and diagnostically promising, yet not uniformly validated or standardized.

In recent years, periodontal diagnostics has moved beyond single inflammatory proteins toward nucleic acids, extracellular vesicles, and broader omics-based approaches. These signals can provide a deeper look at disease activity and host response than traditional markers, and in some cases hint at broader systemic involvement. In practice, however, their use is still limited by how variable sampling can be, how differently assays are performed between studies, and the lack of solid external validation. As a result, translation into routine care continues to depend on reproducible protocols and stronger clinical evidence [2,4].

#### 3.2.1. microRNA Signatures

MicroRNAs are stable regulatory RNAs detectable in gingival crevicular fluid and saliva, with growing diagnostic relevance in periodontitis [26,27,28]. A systematic review of GCF miRNA studies identified miR-146a, miR-200b, miR-223, miR-23a, and miR-203 as most frequently investigated. All except miR-203 demonstrated acceptable diagnostic performance for periodontitis [26]. Meta-analytic evidence highlighted miR-142-3p and miR-146a as the most consistent candidates across microarray and RT-PCR analyses [27]. Pilot studies showed miR-155 as the strongest predictor in non-diabetic individuals, with 82.6% accuracy at a cutoff below 8.97 [28]. In contrast, miR-146a was the only reliable predictor in patients with diabetes, achieving 86.1% accuracy at a cutoff of at least 11.04 [28]. Analysis of GCF revealed significant upregulation of miR-103a-3p, miR-23a-3p, miR-15a-5p, and miR-223-3p in periodontitis [29]. Several miRNAs varied significantly according to disease stage, particularly miR-23a-3p, miR-103a-3p, and miR-423-5p [29]. Salivary small extracellular vesicle–associated miRNAs appear especially promising. Three sEV-enriched miRNAs showed excellent discriminatory performance for periodontitis, with an AUC of 0.96 (single pilot study in salivary sEVs; no external validation—requires replication and confounder control) [30]. Notably, these miRNAs were undetectable in whole saliva, underscoring the value of sEV isolation [30]. Replication across populations and careful control of confounders remain essential translational requirements [26,27,30,31].

#### 3.2.2. Cell-Free DNA

Cell-free DNA reflects cellular death and tissue turnover and may integrate local periodontal damage with systemic inflammatory burden [32,33,34]. Cross-sectional data indicate correlations between cfDNA levels in GCF, saliva, and plasma and clinical periodontal parameters [33]. One study demonstrated increasing cfDNA concentrations with disease severity in GCF and saliva. Plasma cfDNA levels were significantly higher in periodontitis than in healthy controls or patients with gingivitis [33]. Circulating cfDNA levels have been shown to increase in parallel with established clinical and inflammatory indicators of periodontal disease and systemic inflammation, including attachment loss and markers such as hs-CRP [34]. Notably, patients with periodontitis and coexisting cardiovascular disease exhibit higher cfDNA concentrations than those with cardiovascular disease alone, suggesting that periodontal inflammation may contribute to overall vascular inflammatory burden [34]. Clinically, cfDNA should be interpreted as a sensitive but non-specific readout of cell injury and inflammation rather than a disease-specific periodontal marker. It is also highly sensitive to pre-analytical variation (sample collection, processing delays, storage conditions, and leukocyte lysis), which can materially alter measured concentrations. For this reason, cfDNA is best positioned as a component of multimarker panels integrated with host-response biomarkers and clinical parameters, rather than as a stand-alone diagnostic test.

From a clinical perspective, these observations point to a possible link between periodontal disease activity and systemic risk. At the same time, cfDNA remains highly sensitive to preanalytical variation and lacks disease specificity, which limits its use as a stand-alone marker. For this reason, cfDNA is best considered within multimarker panels and interpreted against standardized reference frameworks rather than in isolation [4,32,35]. Experimental studies using nanostructured cfDNA scavengers further implicate cfDNA in periodontal pathogenesis [35].

#### 3.2.3. Extracellular Vesicles and Exosomes

Extracellular vesicles, including exosomes, transport proteins and nucleic acids reflecting immune activation and interorgan communication [36,37]. In this section, the primary diagnostic target is host-derived salivary and GCF sEVs and their cargo, as a real-time readout of periodontal immune dysregulation and its systemic links [36,37,38]. In pilot data, three miRNAs enriched in salivary sEVs discriminated periodontitis with an AUC of 0.96 and were detectable in sEVs but not in whole saliva (same pilot sEV study as above; replication/external validation needed) [30]. Likewise, global 5mC hypermethylation in salivary sEVs distinguished periodontitis from health and gingivitis with perfect discrimination [30]. Composite EV metrics also suggested shifts in vesicle biogenesis toward immune-derived and damage-associated vesicle patterns, with negative associations between CD81/Immune Index and disease severity consistent with immune exhaustion in advanced stages [38], and reduced IL-10 supporting impaired regulatory control in severe disease [38].

Saliva may also contain microbial outer membrane vesicles (OMVs); OMV-related signals (e.g., LPS-positive vesicles and enrichment of OMV-producing periopathogens) have been reported alongside periodontitis-associated EV profiles [30,31]. While host EVs and microbial OMVs can be separated analytically using host-EV enrichment (e.g., tetraspanin-based capture) and OMV-directed readouts, such workflows are not yet harmonized for routine clinical deployment. Standardization of pre-analytics, isolation, and characterization remains the main translational bottleneck [2,36,37]. Despite these constraints, salivary and GCF sEVs remain a promising source of dynamic periodontal biomarkers, particularly when interpreted as adjunctive host-response signals within multimodal risk models [37].

#### 3.2.4. Proteomic Signatures

Mass spectrometry–based proteomics enables multidimensional discovery of salivary disease signatures [39,40,41]. Systematic reviews identified recurrent protein candidates but highlighted substantial heterogeneity in sampling and reporting [39,41]. A meta-analysis identified nine consistently expressed candidates across studies. Upregulated proteins included alpha-amylase, serum albumin, complement C3, neutrophil defensin, profilin-1, and S100-P [39]. Downregulated proteins included carbonic anhydrase VI, immunoglobulin J chain, and lactoferrin [39]. The S100 protein family was particularly abundant in chronic periodontitis. S100A8 and S100A9 showed strong associations with inflammatory activity [40]. Salivary S100A8/S100A9 ratios and MMP-8 differentiated periodontal disease groups [40]. Validation studies showed that eight individual salivary proteins achieved bias-corrected accuracy between 78.8% and 86.8% [42]. Predictive performance improved substantially after age adjustment but less so after smoking adjustment [42]. The best-performing panels included MMP-9, S100A8, alpha-1-acid glycoprotein, pyruvate kinase, and age [43]. Targeted SWATH proteomics identified additional candidates with accuracy exceeding 94% after age adjustment [42]. Meta-analytic evidence showed S100A8 achieved an AUC of 0.71 (meta-analysis; substantial heterogeneity in proteomic workflows and sampling/reporting) [41]. The field is shifting toward targeted validation of a limited set of reproducible candidates [39,41].

#### 3.2.5. Microbiological and Functional Profiling (Oral Microbiome)—Longitudinal Prediction and Therapeutic Implications

Although periodontitis is fundamentally a biofilm-driven inflammatory disease, routine diagnostics still rarely incorporate microbial measurements beyond selected indications. Longitudinal studies, however, suggest that quantitative or profile-based microbial assessments may carry predictive information that complements host-response biomarkers. In prospective cohorts, elevated baseline levels of key anaerobic taxa—particularly *Porphyromonas gingivalis* and *Treponema denticola*—have been associated with subsequent periodontal breakdown, supporting the concept that targeted pathogen burden at the deepest sites can inform risk of progression. Beyond targeted qPCR panels, longitudinal sequencing and multi-omics approaches indicate that dysbiosis is not only compositional but also functional, with shifts toward proteolytic, heme/iron acquisition, and other virulence-associated pathways preceding or accompanying clinical deterioration.

Species-level microbiological testing is often of limited clinical value, as many taxa harbour both commensal and virulent genotypes and the subgingival profile largely reflects a pocket-driven ecology. Accordingly, prognostic work is shifting toward strain/virulence and functional signatures (metagenomic/metatranscriptomic) rather than taxonomy alone. Clinically, these readouts are best used adjunctively with host-response biomarkers to flag a persistent high-virulence ecology and potential non-response, not as stand-alone diagnostics.

From a translational standpoint, microbial profiling may contribute to more specific preventive and therapeutic strategies by identifying (i) patients in whom intensified biofilm control and closer recall intervals are warranted, (ii) individuals likely to respond poorly to conventional therapy, and (iii) virulence mechanisms that can be targeted (e.g., protease-driven tissue disruption and immune subversion). Importantly, treatment-response studies demonstrate that clinical improvement is typically accompanied by a reduction in dysbiotic community features, whereas persistent anaerobic signatures may characterize non-responders, underscoring the potential value of microbiome-informed follow-up.

At present, limitations remain substantial: inter-individual variability, site specificity, sampling heterogeneity, and the lack of universally accepted microbial thresholds constrain routine implementation. Accordingly, the most clinically realistic near-term approach is the integration of standardized, quantitative microbial panels and/or validated dysbiosis indices with host-response biomarkers within multimodal risk models, rather than relying on microbiology as a stand-alone diagnostic.

### 3.3. AI Integration and Decision Support

At present, the use of artificial intelligence in periodontal diagnostics is largely focused on combining information that clinicians already work with on a daily basis—clinical findings, radiographs, and selected molecular data. These tools learn patterns from existing datasets rather than replacing clinical assessment. In practice, their strongest performance has been reported in tasks such as identifying alveolar bone loss and supporting disease staging, where structured imaging and clinical inputs are readily available [44,45,46,47,48,49,50,51].

Not all periodontal AI models are at the same translational stage. Imaging-based algorithms (radiographs/CBCT), particularly for alveolar bone loss detection and staging support, are relatively more mature because inputs and labels are more standardized and benchmarking across centres is feasible. In contrast, multimodal models incorporating molecular biomarkers (saliva/GCF omics, EV cargo, microbiome readouts) remain more exploratory due to pre-analytical sensitivity, assay variability, and heterogeneous endpoint definitions; accordingly, they require stricter standardization and prospective validation before routine use.

For clinical translation, AI performance should be judged primarily by external validation and calibration, not by headline AUC alone. Internal cross-validation can overestimate accuracy in homogeneous datasets; the key question is whether performance holds in independent cohorts, across centres, imaging devices, and case-mix. Calibration (agreement between predicted and observed risk) and clinically relevant thresholds should be reported, because miscalibration can be harmful even when discrimination is high. Explainability should be fit for purpose: at minimum, models should provide traceable inputs and outputs that clinicians can interrogate (e.g., saliency/feature attribution), alongside clear failure modes and uncertainty. Finally, deployment requires a regulatory pathway with clinical evaluation and post-market surveillance, including monitoring for data drift when populations, devices, or clinical protocols change. For transparency, where we cite AUC/accuracy values, we now specify dataset size and whether validation was internal or truly external.

Reported performance includes AUC values around 0.91 and accuracy up to 0.93 for Random Forest classifiers (retrospective ML classifiers; validation type/dataset size as per primary report—external validation and calibration required; drift risk) [50,51]. AI supports therapy personalization by integrating comorbidities, biomarker profiles, and treatment history [45,50,51]. These systems may function as clinical decision support tools, especially in population screening and telemedicine settings [45,52]. Key implementation challenges include algorithm transparency, calibration, data drift monitoring, and external validation [48,49,53,54]. Establishing clinically meaningful decision thresholds remains critical. Standardized validation protocols aligned with International Dental Federation guidance are recommended [55]. AI should support, not replace, clinical judgement. Physician oversight and patient data protection must remain central to deployment strategies [45,54,55].

## 4. Discussion

### 4.1. A Convergence Model: Panels, Platforms, and Prediction

**Figure 4:**
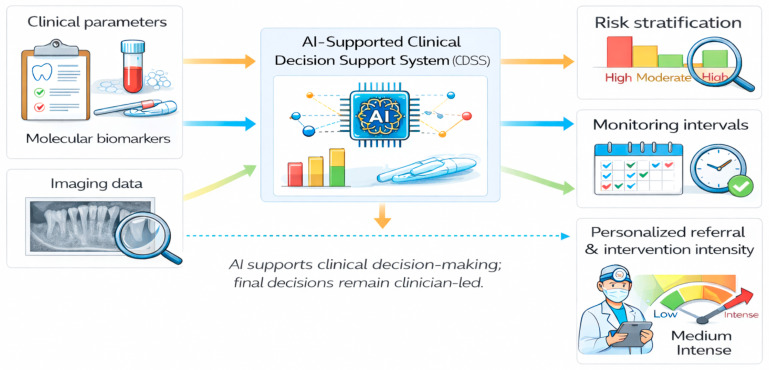
AI-supported clinical decision framework in precision periodontology. Conceptual workflow illustrating how clinical parameters, molecular biomarkers, and imaging data may be integrated through AI-supported clinical decision support systems. The framework highlights clinically actionable outputs, including risk stratification, definition of monitoring intervals, and personalization of referral and intervention intensity. Created in BioRender. Szarpak, L. (2026) https://BioRender.com/fv469x8.

Figure 4 illustrates an AI-supported clinical decision framework integrating clinical data, molecular biomarkers, and imaging to support risk stratification, monitoring strategies, and personalized intervention intensity in precision periodontology. A credible convergence model in precision periodontology rests on synergy across host-response panels, molecular phenotyping, microbiome signatures, and AI-supported decision tools.

From a mechanistic standpoint, the host-response targets discussed here are not generic inflammation markers but reflect defined microbial virulence activities within a dysbiotic biofilm. Key triggers include protease-driven tissue disruption (notably gingipains and related proteolytic ecology), immune subversion with exaggerated neutrophil activation and collagenolysis (captured clinically by aMMP-8 and related protease signatures), and inflammasome-dependent IL-1β signalling that amplifies local damage [4,7]. In parallel, LPS/OMV-associated stimuli and heme/iron acquisition programmes support a persistent pro-inflammatory niche, which is why functional dysbiosis readouts may complement host-response panels. Framing the microbiological layer around virulence mechanisms—rather than taxonomy alone—also aligns with the goal of targeted prevention and treatment intensity.

The most plausible future architecture is an integrated framework. It would enable dynamic assessment of inflammatory activity, identification of disease endotypes, and personalized monitoring and treatment [1,2,4,7,10,45,56,57,58,59,60]. Multiplex point-of-care panels, including MMP-8, calprotectin, and selected cytokines, provide rapid, noninvasive estimates of inflammation and progression risk. Beyond case–control discrimination, a key translational direction is the use of such markers in clinically healthy or minimally inflamed individuals, where elevated aMMP-8 and/or periopathogen signatures may signal increased risk of future attachment loss and justify intensified preventive follow-up. This capability is clinically useful, particularly at the chairside and during follow-up [1,7,58]. Deeper phenotyping layers add biological resolution. Selected microRNAs, cfDNA, and extracellular vesicle features may separate biologically distinct endotypes with different treatment responses and systemic associations [2,4,10,57,59]. Microbiome signatures derived from sequencing and emerging biosensor approaches can capture patterns of dysbiosis and therapy-responsive ecological shifts. They also support monitoring of treatment response and relapse risk [4,10,61]. When these data streams are combined with staging and grading, behavioural risk factors, and comorbidity profiles, AI can support automated risk stratification and progression prediction. It may also help tailor the intensity and timing of interventions [7,45,60].

While host-response biomarkers capture the inflammatory phenotype and tissue susceptibility, longitudinal data suggest that adding a microbial layer may improve prediction of future breakdown, particularly at the site level. A pragmatic approach is a two-axis stratification integrating (i) inflammatory burden (e.g., GCF/circulating host-response panels) with (ii) microbial dysbiosis/selected pathogen load assessed by standardized quantitative assays. Conceptually, patients with a high host-response signal and persistent anaerobic signatures represent a subgroup at highest risk of progression, whereas discordant profiles (high inflammation with low dysbiosis, or vice versa) may indicate distinct biological mechanisms and warrant different preventive intensity. Hence, combined ‘host + microbe’ models should be viewed as complementary rather than competing frameworks, with the most clinically meaningful use in identifying individuals and sites requiring closer monitoring and intensified therapy. For clinical credibility, algorithmic transparency, and external validation are non-negotiable. Interoperability across data sources is equally important if the convergence model is to scale across settings and populations [56,60].

### 4.2. Why Systemic Links Matter Diagnostically

Systemic links matter because periodontitis is increasingly viewed as a condition with whole-body relevance. Periodontal inflammation may reflect existing inflammatory burden. It may also amplify chronic inflammation, increasing the risk of systemic disease onset or progression. Relevant associations include cardiovascular disease, diabetes, COPD, rheumatoid arthritis, and adverse pregnancy outcomes [4,62,63,64,65]. Diabetes is the most clinically actionable oral–systemic link in everyday practice. Observational data support a bidirectional association: poor glycaemic control is associated with greater periodontal inflammation and tissue breakdown, while severe periodontitis is associated with worse glycaemic control and a higher inflammatory burden. Consensus statements and interventional evidence suggest that periodontal therapy can be accompanied by modest improvements in glycaemic measures in people with diabetes, but the relationship should still be framed as an association with biological plausibility rather than definitive causality [66,67]. From a translational standpoint, this supports a pragmatic model in which periodontal screening and longitudinal monitoring are prioritized in patients with diabetes, and where persistent host-response signals may justify closer recall and earlier escalation of supportive care [64,68]. Recent narrative reviews in implant/peri-implant care further underscore the importance of metabolic control and adjunctive strategies in inflammatory oral diseases, reinforcing the practical need for coordinated periodontal–medical management in patients with type 2 diabetes [69]. Most oral–systemic evidence is observational; therefore, associations should not be interpreted as proof of causality, and mechanistic data are presented as biological plausibility rather than definitive evidence.

A practical lesson comes from rheumatoid arthritis, where circulating autoantibodies (ACPA/anti-CCP, often with RF) can precede clinical arthritis by many years—sometimes up to a decade—illustrating that systemic ‘preclinical’ biology is measurable well before overt disease. This precedent supports the rationale for evaluating circulating inflammatory and immune signatures in periodontitis not as diagnostic labels, but as early risk signals that may justify intensified prevention and closer monitoring. In this context, the goal is timely risk stratification and intervention rather than attributing causality from a single biomarker readout.

These links strengthen the case for accurate, scalable detection and monitoring. Early identification of high-risk patients supports integrated management strategies, especially in complex medical patients. In this context, cfDNA and extracellular vesicle cargo are clinically interesting. cfDNA reflects tissue injury and inflammatory intensity. Its levels correlate with periodontitis severity and systemic inflammatory markers, including hs-CRP and periodontal inflamed surface area (PISA) [34,66]. Increased cfDNA has been reported in saliva and in plasma in patients with periodontitis. This pattern supports its potential as a marker of systemic complication risk, particularly cardiovascular risk [34,66]. Extracellular vesicles carry proteins, microRNAs, and other signalling molecules. They reflect immune activation and may facilitate inflammatory cross-talk between organs. EV profiles in saliva and gingival crevicular fluid may therefore track disease activity and provide mechanistic links to systemic inflammation [36,37].

Other systemic candidates deserve brief mention despite the limited longitudinal base [70]. Dysbiosis-derived metabolites, including short-chain fatty acids (SCFAs), may serve as functional readouts of microbial activity with potential links to cardiometabolic and inflammatory pathways [71,72,73]. In parallel, antibody profiles—both autoantibodies and antibodies directed against microbial targets/virulence factors—are attractive because they are analytically stable and may reflect sustained exposure or immune priming; however, their prognostic value in periodontitis-related systemic risk still requires prospective validation.

Incorporating these markers into modern multimarker platforms extends periodontal diagnostics beyond dentistry. It allows simultaneous assessment of periodontal status and systemic inflammatory risk [4,56,64,74,75,76,77,78]. This approach also calls for tighter interdisciplinary collaboration. Integration of dental and medical data is increasingly relevant for improving outcomes in chronic disease pathways [64].

### 4.3. Translational Barriers and Evidence Gaps

Key translational barriers start with heterogeneous case definitions and the absence of harmonized reference standards. These issues undermine comparability and slow clinical validation of new tests [18,64,76]. Frequent reliance on case–control designs inflates performance estimates. It also limits prognostic value in general populations and routine care settings [18]. Sampling variability is another major obstacle. Stimulated versus unstimulated saliva, collection timing, and storage conditions can materially affect analytical results. This variability complicates standardization and replication across centres [39,58,77,78]. Reporting of preanalytical and analytical parameters is often incomplete. Extraction methods, biomarker stability, and the effects of comorbidities are not consistently described. As a result, reproducibility and clinical trust are harder to establish [2,18,58,77]. External validation remains limited in geographically diverse cohorts and in patients with differing comorbidity profiles. This gap constrains implementation in real-world clinical pathways [18,64,76,78]. AI-based tools face additional challenges. Data shift, model calibration, interpretability, and governance of continual learning require explicit handling. Opaque decision processes, overfitting, and limited generalisability remain common barriers to deployment [45,54,79]. Ongoing validation and drift monitoring are therefore required. Ethical standards and interoperability safeguards are also necessary for safe clinical use and durable clinician confidence [54,79].

### 4.4. Practical Roadmap for Clinical Implementation

From a clinical perspective, the expanding landscape of proposed periodontal biomarkers and diagnostic platforms necessitates a pragmatic distinction between tools that may already support patient management and those that, despite biological plausibility, are not yet suitable for routine care. The framework presented here reflects current evidence, translational maturity, and real-world feasibility rather than analytical performance alone.

In practice, personalisation is currently driven by risk-based staging and grading, site-level reassessment (pocket depth, bleeding on probing, residual calculus), and individualized supportive periodontal therapy with recall intervals adjusted to response and risk profile. Modifiable determinants—smoking, diabetes control, adherence, and plaque control capacity—remain the core levers for tailored prevention and maintenance. In selected patients (e.g., rapid progression, incomplete response, recurrent deep sites), adjunctive tools such as point-of-care host-response markers (e.g., aMMP-8) and, where available, targeted microbial/functional readouts may help identify non-responders earlier and justify intensified follow-up.

Host-response point-of-care biomarkers, including active MMP-8, calprotectin, and IL-1β, can already be considered clinically useful as adjunctive tools for screening, risk stratification, and longitudinal monitoring. Mechanistically, these readouts map onto distinct effector arms of periodontal destruction. aMMP-8 reflects neutrophil collagenase activity and is tightly linked to connective tissue breakdown at active sites. Calprotectin (S100A8/A9) is a neutrophil/monocyte-derived alarmin that tracks innate immune activation and local inflammatory burden. IL-1β is an inflammasome-dependent cytokine (including NLRP3 signalling) that amplifies tissue inflammation and supports osteoclast activation, providing a biologically coherent bridge between soft-tissue inflammation and alveolar bone loss. In everyday practice, these tests are most useful as a support to clinical decision-making, particularly in dental settings caring for high-risk or medically compromised patients, such as those with diabetes. In this setting, the clinical objective is not to diagnose diabetes from oral biomarkers, but to support risk-adjusted periodontal prevention and monitoring in a population with higher inflammatory vulnerability and more frequent non-response. They are not meant to replace a full periodontal examination, but rather to add another layer of information to what is already obtained clinically and radiographically. Before wider adoption, their value needs to be confirmed in well-designed clinical studies, using consistent sampling approaches and outcome measures that are meaningful at the chairside. Reliable performance across different clinical settings also remains essential. In contrast, emerging omics-based approaches—including selected microRNAs, cell-free DNA, extracellular vesicles, proteomic profiling, and microbiome analyses—should follow a more cautious translational pathway. At present, these modalities are best suited to confirmatory testing, biological endotyping, and mechanistic exploration in research settings or specialist reference centres. Routine clinical adoption will require validation in large, diverse cohorts, along with standardized sampling procedures and transparent reporting of preanalytical and analytical variables to ensure reproducibility and comparability across centres. In routine care, such approaches should complement point-of-care panels only when deeper biological characterization is clinically justified.

The prominence of aMMP-8 reflects translational maturity rather than preferential selection. It maps to a clear effector pathway (neutrophil collagenase activity), can be implemented in threshold-based point-of-care formats, and has been evaluated more extensively than most candidate markers. By contrast, many alternative biomarkers remain constrained by assay and pre-analytical variability, non-harmonized cut-offs, small or highly selected cohorts, limited external/longitudinal validation, and uncertain incremental value beyond standard clinical staging and grading.

From an implementation perspective, microbiological testing should not be positioned as a stand-alone diagnostic, but as an adjunct in selected scenarios where it can change management: (i) patients with rapid or unexpected progression, (ii) poor or incomplete response after adequate mechanical therapy, (iii) recurrent deep sites despite good plaque control, and (iv) cases in which targeted antimicrobial strategies are considered. In these settings, standardized sampling (deepest sites, avoidance of contamination), quantitative readouts, and a limited panel reflecting dysbiosis or key virulence-associated taxa can be paired with host-response markers to guide recall intervals, reinforce behavioural prevention, and identify non-responders early. Importantly, the clinical goal is not to ‘treat a microbe’ but to use microbial signatures as a proxy for virulence mechanisms and persistence of dysbiosis, thereby supporting personalized preventive and therapeutic intensity.

AI-based decision support systems warrant the highest evidentiary threshold before clinical deployment. While integration of clinical parameters, imaging, and molecular data holds clear promise for personalized periodontal care, such systems should function strictly as clinical decision support tools rather than autonomous diagnostic solutions. Their implementation should be contingent on transparent demonstration of clinical benefit in prospective, multicentre studies with clearly defined endpoints. Algorithm interpretability, software version tracking, and data-drift monitoring are essential, alongside compliance with applicable regulatory frameworks, including the EU AI Act and FDA requirements. Post-market surveillance, including adverse event reporting, model updating, and audits for data protection and ethical compliance, remains critical to maintain clinical trust and patient safety.

In practice, new diagnostic tools need to be introduced gradually. Some host-response biomarkers are already useful for screening and follow-up, but more complex molecular tests and AI-based systems still need a controlled setting before they can be used more widely. Taking this slower approach makes it possible to move forward without putting patient safety at risk and better reflects how periodontal care is actually delivered day-to-day.

### 4.5. Clinical Take-Home: When Host-Response POC Panels Can Change Management

In routine care, host-response point-of-care panels are most useful when they alter the intensity or timing of prevention and follow-up. Practical scenarios include: (1) high-risk screening in smokers, patients with diabetes or other systemic inflammatory comorbidity, and individuals with a history of rapid progression; (2) post-therapy monitoring after non-surgical periodontal therapy to support early identification of incomplete response at residual deep sites; (3) detection of potential non-responders despite adequate instrumentation and plaque control, prompting earlier re-evaluation, reinforcement of behavioural measures, and adjustment of supportive periodontal therapy intervals; and (4) risk-adjusted maintenance planning, where persistently elevated host-response signals can justify closer recall and targeted site-level intervention even when symptoms are limited. These tests are not intended to replace a full periodontal examination; they are best interpreted as adjunctive readouts integrated with staging/grading, clinical parameters, and radiographic findings.

To support clinical decision-making, the following table summarizes which diagnostic tools may be considered for routine use, which remain investigational, and which are not yet appropriate for clinical care (Table 1).

### 4.6. Limitations

As a state-of-the-art narrative review, this work emphasizes recent advances and translational concepts rather than exhaustive evidence synthesis. Selection bias cannot be fully excluded. The included studies may not represent the entire rapidly evolving literature. To mitigate this risk, structured searches of major databases were performed. Backward and forward citation tracking was also applied. Priority was given to recent human studies, clinical validation reports, and consensus documents with clinical relevance.

Study heterogeneity represents an additional limitation. Considerable variability exists in disease definitions, staging and grading frameworks, and sampling protocols. Differences also affect analytical platforms and reported performance metrics. This heterogeneity precluded quantitative pooling and limited direct comparison across technologies and biomarker classes. Diagnostic performance estimates should therefore be considered indicative rather than definitive. Many primary studies were exploratory or cross-sectional.

Case–control designs were common. External validation, longitudinal follow-up, and assessment in comorbid populations were frequently lacking. Such designs are known to inflate diagnostic accuracy and limit generalizability to routine practice. In many studies, basic practical details are still missing. How samples were handled, how long they were stored, or how assays were standardized are often unclear, which makes it difficult to judge how reproducible the results really are. This uncertainty continues to limit meaningful clinical interpretation. To support reproducibility and comparability, a concise minimum reporting checklist for periodontal biomarker studies (preanalytical, analytical, and clinical domains) is provided in Supplementary Table S4.

From a translational standpoint, reproducibility depends as much on pre-analytics as on the biomarker itself. For saliva-based assays, we recommend a standardized protocol specifying unstimulated vs. stimulated collection (unstimulated preferred for comparability), time of day, fasting status, avoidance of toothbrushing/smoking immediately prior, and rapid processing (kept on ice, centrifuged promptly, aliquoted, and stored at −80 °C with freeze–thaw minimized); if stimulated saliva is used, the stimulation method must be reported because it can alter molecular profiles. For GCF, site-specific sampling should be performed with standardized paper strips (e.g., PerioPaper) for a fixed dwell time (commonly ~30 s), with discarding of samples contaminated by blood/saliva and clear reporting of site selection and volume/normalization strategy. For EV-based assays, workflows should follow ISEV MISEV2018/MISEV2023 recommendations, including explicit reporting of isolation method (e.g., SEC/ultracentrifugation/affinity capture), particle and protein quantification, and EV marker characterization; polymer precipitation-only approaches should be interpreted cautiously for biomarker claims. Finally, assay platforms (singleplex ELISA, multiplex bead arrays, lateral-flow/POC tests) should report analytical sensitivity/LOQ, calibration, and inter-assay precision to support comparability across studies.

The situation is similar for AI-based approaches. Most reports are based on retrospective data, small cohorts, or internal validation only, with relatively little experience from prospective or real-world clinical use. As a result, the evidence base remains preliminary, and translation into everyday practice should be approached with caution. Regulatory and implementation issues are therefore discussed conceptually rather than supported by high-level evidence. Overall, these limitations highlight the need for harmonized case definitions. Standardized sampling and reporting protocols are essential. Prospective multicentre studies and rigorous external validation are required to enable reliable clinical translation.

## 5. Conclusions

Advanced periodontal diagnostics are reaching maturity, as multiplex point-of-care biosensors, connected microfluidics, and early wearables enable rapid host-response assessment in saliva and gingival crevicular fluid. In parallel, microRNAs, cell-free DNA, extracellular vesicle–derived signals, proteomic profiles, and microbiome signatures add biological resolution and support integrated oral–systemic risk assessment. Near-term clinical value is greatest for adjunctive risk stratification and longitudinal monitoring, not for replacing conventional periodontal examination. Meaningful clinical and public-health impact will require standardized disease definitions, harmonized sampling and reporting, and validation across diverse populations and comorbidity strata. Regulatory-grade evidence is also needed for in vitro diagnostics and artificial intelligence software classified as medical devices.

## Figures and Tables

**Table 1:** Translational roadmap for advanced periodontal diagnostics: examples, indicative performance, limitations, and recommended clinical use.

Category	Representative Examples	Indicative Performance (AUC/Sens–Spec)	Main Limitations	Recommended Clinical Use
**Conventional** **clinical assessment**	Probing depth, BOP, CAL, radiographs; staging/grading	N/A (reference standard)	Examiner variability; low sensitivity for “activity”	Routine baseline + monitoring
**POC host-response biomarkers** **(saliva/GCF)**	aMMP-8, calprotectin, IL-1β; multiplex panels	AUC ~0.70–0.90 (pooled); sens/spec variable	Case–control spectrum bias; thresholds/assay heterogeneity	Adjunct for screening/monitoring; non-responders
**POC biosensor** **platforms**	Lateral-flow, lab-on-chip immunoassays; SAW biosensors	Single-study AUC ~0.8–0.86 (platform-dependent)	External validation; matrix effects; calibration	Selected settings; implementation pilots
**miRNA panels** **(saliva/GCF)**	miRNA signatures (e.g., miR-155/miR-146a, etc.)	Often high in single studies; limited pooled evidence	Platform variability; confounding; limited external validation	Research/selected risk stratification
**cfDNA/methylation**	cfDNA burden; 5mC patterns	Mostly exploratory; few robust metrics	Pre-analytics critical; specificity	Research/mechanistic endotyping
**EVs/exosomes** **(host sEV cargo)**	sEV miRNA; EV indices; EV methylation	Very high in pilots (e.g., AUC 0.96)	EV isolation standardization; OMV contamination	Research/reference centres
**Proteomics**	Salivary MS panels; recurrent inflammatory proteins	Mixed; some validated signals; workflow-dependent	Standardization, cost, multi-centre replication	Reference labs; biomarker discovery translation
**Microbiome/** **functional profiling**	qPCR pathogen burden; dysbiosis indices; metagenomics	Limited longitudinal/predictive metrics	Strain variability; ecology confounding	Adjunct in selected scenarios; research
**AI decision support**	Imaging-based staging; multimodal risk prediction	AUC ~0.9 in retrospective reports	External validation; calibration; drift; regulation	Decision support after validation; controlled rollout

Legend: AI, artificial intelligence; AUC, area under the receiver operating characteristic curve; BOP, bleeding on probing; CAL, clinical attachment level; cfDNA, cell-free DNA; EV, extracellular vesicle; GCF, gingival crevicular fluid; IL-1β, interleukin-1 beta; miRNA, microRNA; aMMP-8, active matrix metalloproteinase-8; MS, mass spectrometry; OMV, outer membrane vesicle; POC, point-of-care; qPCR, quantitative polymerase chain reaction; SAW, surface acoustic wave; sEV, small extracellular vesicle.

## Data Availability

No new data were created or analyzed in this study.

## Supplementary Materials

The following supporting information can be downloaded at: https://www.mdpi.com/article/10.3390/jcm15031142/s1, Supplementary Table S1: Full database search strategies; Supplementary Table S2: Practical inclusion/exclusion criteria applied in this narrative review; Supplementary Table S3: Evidence maturity of diagnostic domains and key performance claims; Supplementary Table S4. Minimum reporting checklist for periodontal biomarker studies.

## Abbreviations

The following abbreviations are used in this manuscript:

AI	Artificial Intelligence
AUC	Area Under the Curve
COPD	Chronic Obstructive Pulmonary Disease
EV	Extracellular Vesicle
GCF	Gingival Crevicular Fluid
MMP	Matrix Metalloproteinase
PISA	Periodontal Inflamed Surface Area
POC	Point-of-Care
RT-PCR	Reverse Transcription Polymerase Chain Reaction

## References

[1] Korgaonkar J., Tarman A.Y., Ceylan Koydemir H., Chukkapalli S.S. (2024). Periodontal disease and emerging point-of-care technologies for its diagnosis. Lab Chip.

[2] Dolińska E., Wiśniewski P., Pietruska M. (2024). Periodontal Molecular Diagnostics: State of Knowledge and Future Prospects for Clinical Application. Int. J. Mol. Sci..

[3] Korte D.L., Kinney J. (2016). Personalized medicine: An update of salivary biomarkers for periodontal diseases. Periodontology 2000.

[4] Foroughi M., Torabinejad M., Angelov N., Ojcius D.M., Parang K., Ravnan M., Lam J. (2025). Bridging oral and systemic health: Exploring pathogenesis, biomarkers, and diagnostic innovations in periodontal disease. Infection.

[5] Liccardo D., Cannavo A., Spagnuolo G., Ferrara N., Cittadini A., Rengo C., Rengo G. (2019). Periodontal Disease: A Risk Factor for Diabetes and Cardiovascular Disease. Int. J. Mol. Sci..

[6] Jepsen S., Kebschull M., Deschner J. (2011). Relationship between periodontitis and systemic diseases. Bundesgesundheitsblatt-Gesundheitsforschung-Gesundheitsschutz.

[7] Räisänen I.T., Penttala M., Sahni V., Toby Thomas J., Grigoriadis A., Sakellari D., Gupta S., Pärnänen P., Pätilä T., Sorsa T. (2025). Combining oral fluid aMMP-8, calprotectin, and CCAAs in dental panoramic radiography for periodontal disease and systemic disease risk assessment: A point-of-care diagnostic approach. Expert Rev. Mol. Diagn..

[8] Zhang W., Du J., Wang K., Li Y., Chen C., Yang L., Kan Z., Dong B., Wang L., Xu L. (2023). Integrated dual-channel electrochemical immunosensor for early diagnosis and monitoring of periodontitis by detecting multiple biomarkers in saliva. Anal. Chim. Acta.

[9] Steigmann L., Maekawa S., Sima C., Travan S., Wang C.W., Giannobile W.V. (2020). Biosensor and Lab-on-a-chip Biomarker-identifying Technologies for Oral and Periodontal Diseases. Front. Pharmacol..

[10] Ashraf M.U., Mustaffa K.M.F., Hasan N.W.M., Memon M.A., Butt D.Q., Mahmood R., Shahidan W.N.S. (2025). Beyond traditional diagnosis: Aptamer-based microRNA detection for the early diagnosis of periodontitis. Mol. Biol. Rep..

[11] Nozawa A., Oshima H., Togawa N., Nozaki T., Murakami S. (2020). Development of Oral Care Chip, a novel device for quantitative detection of the oral microbiota associated with periodontal disease. PLoS ONE.

[12] Saikumar J., Ramachandran K., Vaidya V.S. (2014). Noninvasive micromarkers. Clin. Chem..

[13] Faruq O., Vecchione A. (2015). microRNA: Diagnostic Perspective. Front. Med..

[14] Huang W. (2017). MicroRNAs: Biomarkers, Diagnostics, and Therapeutics. Bioinformatics in MicroRNA Research.

[15] Umeizudike K.A., Lähteenmäki H., Räisänen I.T., Taylor J.J., Preshaw P.M., Bissett S.M., Tervahartiala T., Nwhator S.O., Pärnänen P., Sorsa T. (2022). Ability of matrix metalloproteinase-8 biosensor, IFMA, and ELISA immunoassays to differentiate between periodontal health, gingivitis, and periodontitis. J. Periodontal Res..

[16] Guida L., Bencivenga D., Annunziata M., Arcadio F., Borriello A., Della Ragione F., Formisano A., Piccirillo A., Zeni L., Cennamo N. (2023). An optical fiber-based point-of-care test for periodontal MMP-8 detection: A proof of concept. J. Dent..

[17] Joe C., Lee B.H., Kim S.H., Ko Y., Gu M.B. (2022). Aptamer duo-based portable electrochemical biosensors for early diagnosis of periodontal disease. Biosens. Bioelectron..

[18] Rakic M., Calciolari E., Grant M.M., Radovanovic S., Bostanci N., Preshaw P.M. (2025). Host Markers of Periodontal Diseases: Meta-Analysis of Diagnostic Accuracy Studies. J. Clin. Periodontol..

[19] Arias-Bujanda N., Regueira-Iglesias A., Balsa-Castro C., Nibali L., Donos N., Tomás I. (2020). Accuracy of single molecular biomarkers in saliva for the diagnosis of periodontitis: A systematic review and meta-analysis. J. Clin. Periodontol..

[20] Domokos Z., Simon F., Uhrin E., Szabó B., Váncsa S., Varga G., Hegyi P., Kerémi B., Németh O. (2024). Evaluating salivary MMP-8 as a biomarker for periodontal diseases: A systematic review and meta-analysis. Heliyon.

[21] Zhang D., Xu C., Liang M., Shao W., Wang P., Yang Y., Guo K. (2025). Diagnostic Accuracy of Matrix Metalloproteinase-8 for Detecting Periodontal Disease: A Meta-Analysis. Oral Dis..

[22] Jeon S., Kim S.H., Heo G., Heo H.J., Chae S.Y., Kwon Y.W., Lee S.K., Han D.W., Kim H.J., Kim Y.H. (2025). A Wearable Electrochemical Biosensor for Salivary Detection of Periodontal Inflammation Biomarkers: Molecularly Imprinted Polymer Sensor with Deep Learning Integration. Adv. Sci..

[23] Pan J., Li X., Sun R., Xu Y., Shi Z., Dai C., Wen H., Han R.P.S., Ye Q., Zhang F. (2024). Hydrogel-based radio frequency H2S sensor for in situ periodontitis monitoring and antibacterial treatment. Biosens. Bioelectron..

[24] de Almeida E., Bueno L., Kwong M.T., Bergmann J.H.M. (2023). Performance of Oral Cavity Sensors: A Systematic Review. Sensors.

[25] Wang J., Yu J., Wang T., Li C., Wei Y., Deng X., Chen X. (2020). Emerging intraoral biosensors. J. Mater. Chem. B.

[26] Cosín-Villanueva M., Almiñana-Pastor P.J., García-Giménez J.L., López-Roldán A. (2024). Study of microRNAs in Gingival Crevicular Fluid as Periodontal Diseases Biomarkers: Systematic Review. Int. J. Mol. Sci..

[27] Asaad F., Garaicoa-Pazmiño C., Dahlin C., Larsson L. (2020). Expression of MicroRNAs in Periodontal and Peri-Implant Diseases: A Systematic Review and Meta-Analysis. Int. J. Mol. Sci..

[28] Al-Rawi N.H., Al-Marzooq F., Al-Nuaimi A.S., Hachim M.Y., Hamoudi R. (2020). Salivary microRNA 155, 146a/b and 203: A pilot study for potentially non-invasive diagnostic biomarkers of periodontitis and diabetes mellitus. PLoS ONE.

[29] Costantini E., Sinjari B., Di Giovanni P., Aielli L., Caputi S., Muraro R., Murmura G., Reale M. (2023). TNFα, IL-6, miR-103a-3p, miR-423-5p, miR-23a-3p, miR-15a-5p and miR-223-3p in the crevicular fluid of periodontopathic patients correlate with each other and at different stages of the disease. Sci. Rep..

[30] Han P., Bartold P.M., Salomon C., Ivanovski S. (2020). Salivary Small Extracellular Vesicles Associated miRNAs in Periodontal Status—A Pilot Study. Int. J. Mol. Sci..

[31] Buragaite-Staponkiene B., Rovas A., Puriene A., Snipaitiene K., Punceviciene E., Rimkevicius A., Butrimiene I., Jarmalaite S. (2023). Gingival Tissue MiRNA Expression Profiling and an Analysis of Periodontitis-Specific Circulating MiRNAs. Int. J. Mol. Sci..

[32] Viglianisi G., Santonocito S., Polizzi A., Troiano G., Amato M., Zhurakivska K., Pesce P., Isola G. (2023). Impact of Circulating Cell-Free DNA (cfDNA) as a Biomarker of the Development and Evolution of Periodontitis. Int. J. Mol. Sci..

[33] Zhu X., Chu C.J., Pan W., Li Y., Huang H., Zhao L. (2022). The Correlation between Periodontal Parameters and Cell-Free DNA in the Gingival Crevicular Fluid, Saliva, and Plasma in Chinese Patients: A Cross-Sectional Study. J. Clin. Med..

[34] Isola G., Polizzi A., Mascitti M., Santonocito S., Ronsivalle V., Cicciù M., Pesce P. (2023). Impact of periodontitis on circulating cell-free DNA levels as a measure of cardiovascular disease. Clin. Oral Investig..

[35] Huang H., Pan W., Wang Y., Kim H.S., Shao D., Huang B., Ho T.C., Lao Y.H., Quek C.H., Shi J. (2022). Nanoparticulate cell-free DNA scavenger for treating inflammatory bone loss in periodontitis. Nat. Commun..

[36] Cai R., Wang L., Zhang W., Liu B., Wu Y., Pang J., Ma C. (2023). The role of extracellular vesicles in periodontitis: Pathogenesis, diagnosis, and therapy. Front. Immunol..

[37] Han P., Bartold P.M., Ivanovski S. (2022). The emerging role of small extracellular vesicles in saliva and gingival crevicular fluid as diagnostics for periodontitis. J. Periodontal Res..

[38] Bonilla M., Bravo M., Peñalver I., Mesa F. (2025). Exosome profile and composite indices reflect immune exhaustion in periodontitis. Sci. Rep..

[39] Hu H., Leung W.K. (2023). Mass Spectrometry-Based Proteomics for Discovering Salivary Biomarkers in Periodontitis: A Systematic Review. Int. J. Mol. Sci..

[40] Sánchez-Medrano A.G., Martinez-Martinez R.E., Soria-Guerra R., Portales-Perez D., Bach H., Martinez-Gutierrez F. (2023). A systematic review of the protein composition of whole saliva in subjects with healthy periodontium compared with chronic periodontitis. PLoS ONE.

[41] Corana M., Baima G., Iaderosa G., Franco F., Zhang J., Berta G.N., Romano F., Aimetti M. (2025). Salivary Proteomics for Detecting Novel Biomarkers of Periodontitis: A Systematic Review. J. Periodontal Res..

[42] Blanco-Pintos T., Regueira-Iglesias A., Relvas M., Alonso-Sampedro M., Bravo S.B., Balsa-Castro C., Tomás I. (2025). Diagnostic Accuracy of Novel Protein Biomarkers in Saliva to Detect Periodontitis Using Untargeted ‘SWATH’ Mass Spectrometry. J. Clin. Periodontol..

[43] Grant M.M., Taylor J.J., Jaedicke K., Creese A., Gowland C., Burke B., Doudin K., Patel U., Weston P., Milward M. (2022). Discovery, validation, and diagnostic ability of multiple protein-based biomarkers in saliva and gingival crevicular fluid to distinguish between health and periodontal diseases. J. Clin. Periodontol..

[44] Roy R., Chopra A., Karmakar S., Bhat S.G. (2025). Applications of Artificial Intelligence (AI) for Diagnosis of Periodontal/Peri-Implant Diseases: A Narrative Review. J. Oral Rehabil..

[45] Pitchika V., Büttner M., Schwendicke F. (2024). Artificial intelligence and personalized diagnostics in periodontology: A narrative review. Periodontology 2000.

[46] Revilla-León M., Gómez-Polo M., Barmak A.B., Inam W., Kan J.Y.K., Kois J.C., Akal O. (2023). Artificial intelligence models for diagnosing gingivitis and periodontal disease: A systematic review. J. Prosthet. Dent..

[47] Patil S., Joda T., Soffe B., Awan K.H., Fageeh H.N., Tovani-Palone M.R., Licari F.W. (2023). Efficacy of artificial intelligence in the detection of periodontal bone loss and classification of periodontal diseases: A systematic review. J. Am. Dent. Assoc..

[48] Suh B., Yu H., Cha J.K., Choi J., Kim J.W. (2025). Explainable Deep Learning Approaches for Risk Screening of Periodontitis. J. Dent. Res..

[49] Khubrani Y.H., Thomas D., Slator P.J., White R.D., Farnell D.J.J. (2025). Detection of periodontal bone loss and periodontitis from 2D dental radiographs via machine learning and deep learning: Systematic review employing APPRAISE-AI and meta-analysis. Dentomaxillofac. Radiol..

[50] Rebeiz T., Lawand G., Martin W., Gonzaga L., Revilla-León M., Khalaf S., Megarbané J.M. (2025). Development of an artificial intelligence model for assisting periodontal therapy decision-making: A retrospective longitudinal cohort study. J. Dent..

[51] Furquim C.P., Caruth L., Chandrasekaran G., Cucchiara A., Kallan M.J., Martin L., Feres M., Bittinger K., Divaris K., Glessner J. (2025). Developing Predictive Models for Periodontitis Progression Using Artificial Intelligence: A Longitudinal Cohort Study. J. Clin. Periodontol..

[52] Tao L.R., Li Y., Wu X.Y., Gu Y., Xie Y., Yu X.Y., Lai H.C., Tonetti M.S. (2025). Deep Learning Photo Processing for Periodontitis Screening. J. Dent. Res..

[53] Farooqi O.A., Fru G.A., Gong Y.M., Oswald L.E., DeNucci D.J. (2025). Evaluation of artificial intelligence-based clinical decision support systems for caries and periodontal bone loss: An external validation study. J. Am. Dent. Assoc..

[54] Schwendicke F., Samek W., Krois J. (2020). Artificial Intelligence in Dentistry: Chances and Challenges. J. Dent. Res..

[55] Tuygunov N., Samaranayake L., Khurshid Z., Rewthamrongsris P., Schwendicke F., Osathanon T., Yahya N.A. (2025). The Transformative Role of Artificial Intelligence in Dentistry: A Comprehensive Overview Part 2: The Promise and Perils, and the International Dental Federation Communique. Int. Dent. J..

[56] Herrera D., Tonetti M.S., Chapple I., Kebschull M., Papapanou P.N., Sculean A., Abusleme L., Aimetti M., Belibasakis G., Blanco J. (2025). Consensus Report of the 20th European Workshop on Periodontology: Contemporary and Emerging Technologies in Periodontal Diagnosis. J. Clin. Periodontol..

[57] Bostanci N., Belibasakis G.N. (2023). Precision periodontal care: From omics discoveries to chairside diagnostics. Clin. Oral Investig..

[58] Alavi S.E., Sharma L.A., Sharma A., Ebrahimi Shahmabadi H. (2025). Salivary Biomarkers in Periodontal Disease: Revolutionizing Early Detection and Precision Dentistry. Mol. Diagn. Ther..

[59] Divaris K., Moss K., Beck J.D. (2020). Biologically informed stratification of periodontal disease holds the key to achieving precision oral health. J. Periodontol..

[60] Mörch C.M., Atsu S., Cai W., Li X., Madathil S.A., Liu X., Mai V., Tamimi F., Dilhac M.A., Ducret M. (2021). Artificial Intelligence and Ethics in Dentistry: A Scoping Review. J. Dent. Res..

[61] Usui M., Miyagi S., Yamanaka R., Oka Y., Kobayashi K., Sato T., Sano K., Onizuka S., Inoue M., Fujii W. (2025). Measuring the Invisible: Microbial Diagnostics for Periodontitis—A Narrative Review. Int. J. Mol. Sci..

[62] Hajishengallis G., Chavakis T. (2021). Local and systemic mechanisms linking periodontal disease and inflammatory comorbidities. Nat. Rev. Immunol..

[63] Kalhan A.C., Wong M.L., Allen F., Gao X. (2022). Periodontal disease and systemic health: An update for medical practitioners. Ann. Acad. Med. Singap..

[64] Herrera D., Sanz M., Shapira L., Brotons C., Chapple I., Frese T., Graziani F., Hobbs F.D.R., Huck O., Hummers E. (2023). Association between periodontal diseases and cardiovascular diseases, diabetes and respiratory diseases: Consensus report of the Joint Workshop by the European Federation of Periodontology (EFP) and the European arm of the World Organization of Family Doctors (WONCA Europe). J. Clin. Periodontol..

[65] Hajishengallis G. (2022). Interconnection of periodontal disease and comorbidities: Evidence, mechanisms, and implications. Periodontology 2000.

[66] Cosola S., Butera A., Hailu Zergaw A., George J., Covani U., Arrighi A., Toti P., Scribante A., Menchini-Fabris G.B. (2025). Glycemic Control and Implant Stability in Patients with Type II Diabetes: Narrative Review. Healthcare.

[67] Butera A., Maiorani C., Gallo S., Pascadopoli M., Venugopal A., Marya A., Scribante A. (2022). Evaluation of Adjuvant Systems in Non-Surgical Peri-Implant Treatment: A Literature Review. Healthcare.

[68] Chapple I.L., Genco R., Working Group 2 of Joint EFP/AAP Workshop (2013). Diabetes and periodontal diseases: Consensus report of the Joint EFP/AAP Workshop on Periodontitis and Systemic Diseases. J. Clin. Periodontol..

[69] Herrera D., Sanz M., Shapira L., Brotons C., Chapple I., Frese T., Graziani F., Hobbs F.D.R., Huck O., Hummers E. (2024). Periodontal diseases and cardiovascular diseases, diabetes, and respiratory diseases: Summary of the consensus report by the European Federation of Periodontology and WONCA Europe. Eur. J. Gen. Pract..

[70] Mohsenzadeh A., Pourasgar S., Mohammadi A., Nazari M., Nematollahi S., Karimi Y., Firoozbakhsh P., Mohsenzadeh H., Kamali K., Elahi R. (2025). The gut microbiota and cardiovascular disease: Exploring the role of microbial dysbiosis and metabolites in pathogenesis and therapeutics. Life Sci..

[71] de Ferranti S.D., Teng J.E., Arslanian S.A., Atz A.M., Brothers J.A., Cartoski M.J., Freemon D.R., Magge S.N., Mahle W.T., Mietus-Snyder M. (2025). A multicenter trial to test pitavastatin calcium in youth with combined dyslipidemia of obesity: Design, implementation, challenges, and responses. Am. Heart J..

[72] Takeuchi-Hatanaka K., Shirahase Y., Yoshida T., Kono M., Toya N., Konishi K., Omori K., Takashiba S. (2025). Salivary short chain fatty acids serve as biomarkers of periodontal inflammatory burden. Sci. Rep..

[73] Amato M., Polizzi A., Viglianisi G., Leonforte F., Mascitti M., Isola G. (2025). Impact of Periodontitis and Oral Dysbiosis Metabolites in the Modulation of Accelerating Ageing and Human Senescence. Metabolites.

[74] Martínez-García M., Hernández-Lemus E. (2021). Periodontal Inflammation and Systemic Diseases: An Overview. Front. Physiol..

[75] Mattos M.C.O., Vivacqua A., Carneiro V.M.A., Grisi D.C., Guimarães M.D.C.M. (2025). Interaction of the Systemic Inflammatory State, Inflammatory Mediators, and the Oral Microbiome. Oral Microbiome.

[76] Bostanci N., Manoil D., Van Holm W., Belibasakis G.N., Teughels W. (2025). Microbial Markers for Diagnosis and Risk Assessment for Periodontal Diseases: A Systematic Literature Search and Narrative Synthesis. J. Clin. Periodontol..

[77] Gürsoy U.K., Kantarci A. (2022). Molecular biomarker research in periodontology: A roadmap for translation of science to clinical assay validation. J. Clin. Periodontol..

[78] Ebersole J.L., Hasturk H., Huber M., Gellibolian R., Markaryan A., Zhang X.D., Miller C.S. (2024). Realizing the clinical utility of saliva for monitoring oral diseases. Periodontology 2000.

[79] Shan T., Tay F.R., Gu L. (2021). Application of Artificial Intelligence in Dentistry. J. Dent. Res..

