# Supplementary material for: Advanced Diagnostic Technologies and Molecular Biomarkers in Periodontitis: Systemic Health Implications and Translational Perspectives
Source: J Clin Med. 2026 Feb 2;15(3):1142. doi: 10.3390/jcm15031142 (PMC12898585; doi:10.3390/jcm15031142)
Supplement: Supplementary file 1 [file jcm-15-01142-s001.zip › Supplementary Table S1.pdf]

Supplemenaty Table S1. Full database search strategies.

PubMed / MEDLINE (via PubMed)
(<br>"Periodontitis"[Mesh] OR "Periodontal Diseases"[Mesh] OR periodontitis[tiab] OR "periodontal disease*"[tiab] OR "periodontal inflammation"[tiab]<br>)<br>AND<br>(<br>/* A. Point-of-care, biosensors, platforms */<br>"Point-of-Care Systems"[Mesh] OR "Biosensing Techniques"[Mesh] OR point-of-care[tiab] OR "point of care"[tiab] OR POCT[tiab] OR chairside[tiab] OR bedside[tiab]<br>OR biosensor*[tiab] OR immunosensor*[tiab] OR "lab-on-a-chip"[tiab] OR "lab on a chip"[tiab] OR microfluidic*[tiab] OR "lateral flow"[tiab]<br>OR electrochemical[tiab] OR voltammetric[tiab] OR plasmonic[tiab] OR "surface acoustic wave"[tiab] OR SAW[tiab]<br>OR wearable*[tiab] OR intraoral[tiab] OR "tooth-mounted"[tiab] OR mouthguard[tiab]<br>/* B. Host-response / protein biomarkers */<br>OR "Biomarkers"[Mesh] OR biomarker*[tiab] OR "host response"[tiab] OR "host-response"[tiab]<br>OR "Matrix Metalloproteinase 8"[tiab] OR MMP-8[tiab] OR aMMP-8[tiab]<br>OR "Interleukin-1beta"[tiab] OR "Interleukin 1 beta"[tiab] OR IL-1β[tiab] OR IL-1beta[tiab]<br>OR calprotectin[tiab] OR S100A8[tiab] OR S100A9[tiab]<br>OR cytokine*[tiab] OR chemokine*[tiab]<br>OR RANKL[tiab] OR OPG[tiab] OR osteoprotegerin[tiab] OR "RANKL/OPG"[tiab]<br>/* C. Nucleic acids / EVs / methylation / proteomics / microbiome */<br>OR "MicroRNAs"[Mesh] OR microRNA*[tiab] OR miRNA*[tiab]<br>OR "cell-free DNA"[tiab] OR cfDNA[tiab]<br>OR "DNA Methylation"[Mesh] OR methylation[tiab] OR "5-methylcytosine"[tiab] OR 5mC[tiab]<br>OR "Exosomes"[Mesh] OR exosome*[tiab] OR "extracellular vesicle*"[tiab] OR EV[tiab] OR EVs[tiab] OR "small extracellular vesicle*"[tiab] OR sEV[tiab] OR sEVs[tiab]<br>OR "Proteomics"[Mesh] OR proteomic*[tiab] OR proteome*[tiab] OR "Mass Spectrometry"[Mesh] OR "mass spectrometry"[tiab] OR SWATH[tiab]<br>OR "Metagenomics"[Mesh] OR metagenomic*[tiab] OR metatranscriptomic*[tiab] OR microbiome[tiab] OR "oral microbiome"[tiab] OR dysbiosis[tiab]<br>OR "16S"[tiab] OR "next generation sequencing"[tiab] OR NGS[tiab] OR qPCR[tiab] OR "quantitative PCR"[tiab]<br>/* D. AI / ML (incl. imaging-based periodontal diagnostics) */<br>OR "Artificial Intelligence"[Mesh] OR "Machine Learning"[Mesh] OR "artificial intelligence"[tiab] OR "machine learning"[tiab] OR "deep learning"[tiab] OR "neural network*"[tiab]<br>OR "computer vision"[tiab] OR radiograph*[tiab] OR "panoramic radiograph*"[tiab] OR CBCT[tiab] OR imaging[tiab]<br>)<br>AND<br>("2018/01/01"[Date - Publication] : "2026/01/10"[Date - Publication])

AND<br>(english[lang])
Scopus
TITLE-ABS-KEY(<br>periodontitis OR "periodontal disease*" OR "periodontal inflammation"<br>)<br>AND<br>TITLE-ABS-KEY(<br>"point-of-care" OR "point of care" OR POCT OR chairside OR bedside<br>OR biosensor* OR immunosensor* OR "lab-on-a-chip" OR "lab on a chip" OR microfluidic* OR "lateral flow"<br>OR electrochemical OR voltammetric OR plasmonic OR "surface acoustic wave" OR SAW<br>OR wearable* OR intraoral OR "tooth-mounted" OR mouthguard<br>OR biomarker* OR "host response" OR "host-response"<br>OR "MMP-8" OR "matrix metalloproteinase 8" OR aMMP-8<br>OR "IL-1beta" OR "IL-1β" OR "interleukin-1 beta" OR calprotectin OR S100A8 OR S100A9<br>OR cytokine* OR chemokine* OR RANKL OR OPG OR osteoprotegerin OR "RANKL/OPG"<br>OR microRNA OR microRNA* OR miRNA OR miRNA*<br>OR "cell-free DNA" OR cfDNA<br>OR methylation OR "DNA methylation" OR "5-methylcytosine" OR 5mC<br>OR exosome* OR "extracellular vesicle*" OR EV OR EVs OR "small extracellular vesicle*" OR sEV OR sEVs<br>OR proteomic* OR proteome* OR "mass spectrometry" OR SWATH<br>OR microbiome OR "oral microbiome" OR dysbiosis OR metagenomic* OR metatranscriptomic* OR "16S" OR NGS OR qPCR OR "quantitative PCR"<br>OR "artificial intelligence" OR "machine learning" OR "deep learning" OR "neural network*" OR "computer vision"<br>OR radiograph* OR "panoramic radiograph*" OR CBCT OR imaging<br>)<br>AND (PUBYEAR > 2017 AND PUBYEAR < 2027)<br>AND (LIMIT-TO(LANGUAGE, "English"))<br>AND (LIMIT-TO(DOCTYPE, "ar") OR LIMIT-TO(DOCTYPE, "re"))
