# Supplementary material for: Advanced Diagnostic Technologies and Molecular Biomarkers in Periodontitis: Systemic Health Implications and Translational Perspectives
Source: J Clin Med. 2026 Feb 2;15(3):1142. doi: 10.3390/jcm15031142 (PMC12898585; doi:10.3390/jcm15031142)
Supplement: Supplementary file 1 [file jcm-15-01142-s001.zip › Supplementary Table S2.pdf]

Supplementary Table S2. Practical inclusion/exclusion criteria applied in this narrative review

Domain	Included (minimum)	Excluded / down-weighted
**Population / setting**	Human studies in periodontal health/gingivitis/periodontitis; key translational/mechanistic work when directly informing clinical interpretation	Non-periodontal populations without clear relevance
**Sample types**	Saliva (stimulated/unstimulated), GCF, blood/serum/plasma, subgingival plaque	Studies without clear sample handling description (pre-analytics not reported)
**POC / biosensors**	POC devices with defined target analytes and clinically interpretable outputs; diagnostic accuracy/monitoring studies with clear reference standard	Pure engineering reports without clinical samples or without performance data in relevant biofluids
**Host-response markers (e.g., aMMP-8, IL-1β, calprotectin)**	Human studies reporting clinical labels and/or activity/progression/response; clear assay description	Small case–control studies interpreted cautiously unless independently replicated
**miRNA / cfDNA / methylation**	Studies with defined periodontal phenotypes and adequate reporting of extraction, normalisation, and analytical platform	Highly exploratory signals without methodological transparency or with high risk of confounding (uncontrolled)
**EVs / exosomes (sEVs)**	Studies reporting isolation approach and basic characterisation; clinically defined phenotypes	EV studies without characterisation/standardisation or with ambiguous separation from microbial vesicles (down-weighted)
**Proteomics**	Human saliva/GCF proteomics with clear workflow and clinically defined groups; preference for validation cohorts	Discovery-only studies without validation; unclear pre-analytics or batch control
**Microbiome / functional profiling**	Human microbiome studies with defined phenotypes; preference for longitudinal/prognostic endpoints and functional/virulence-oriented readouts	Taxonomy-only associations interpreted cautiously due to ecological confounding and strain variability
**AI / machine learning**	Clear outcome labels; transparent validation (held-out test set and/or external validation); methods sufficient to assess leakage and generalisability	Performance claims without appropriate validation or unclear separation of training/testing (excluded); single-centre, highly curated datasets down-weighted unless externally validated
**Study designs (overall weighting)**	Prospective/longitudinal and interventional studies prioritised; cross-sectional included if clinically interpretable	Evidence from small, highly selected case–control designs down-weighted for spectrum bias

		unless supported by independent cohorts/longitudinal outcomes

Abbreviations: AI: artificial intelligence; aMMP-8: active matrix metalloproteinase-8; cfDNA: cell-free DNA; EVs: extracellular vesicles; GCF: gingival crevicular fluid; IL-1β: interleukin-1 beta; miRNA: microRNA; POC: point-of-care; sEVs: small extracellular vesicles.
