# Supplementary material for: Advanced Diagnostic Technologies and Molecular Biomarkers in Periodontitis: Systemic Health Implications and Translational Perspectives
Source: J Clin Med. 2026 Feb 2;15(3):1142. doi: 10.3390/jcm15031142 (PMC12898585; doi:10.3390/jcm15031142)
Supplement: Supplementary file 1 [file jcm-15-01142-s001.zip › Supplementary Table S3.pdf]

Supplementary Table S3. Evidence maturity of diagnostic domains and key performance claims.

Diagnostic domain / tool	Representative readout(s)	Key performance claim(s) cited in manuscript	Evidence source type	Validation status	Maturity grade	Notes relevant to bias/heterogeneity
**POC host-response biomarkers (saliva/GCF)**	MMP-8 / aMMP-8, ± IL-1β, calprotectin	Meta-analyses: AUC ~0.70–0.90; Sens 0.49–0.84; Spec 0.62–0.79	Meta-analysis (diagnostic accuracy)	Pooled evidence across studies	Validated	Marked heterogeneity in case definitions, sampling, and thresholds; spectrum bias common in case–control studies.
**POC biosensor example (single platform)**	Surface acoustic wave MMP-8 biosensor	AUC 0.81, accuracy 74.2% (periodontitis+gingivitis vs health); AUC 0.86, accuracy 82.8% (periodontitis vs health+gingivitis)	Single study/platform report	Typically internal / limited external	Exploratory–Validated (platform-specific)	Good proof-of-concept; generalisability depends on matrix effects, calibration and external replication.
**Chairside thresholded marker**	aMMP-8 (cut-off approach)	Cut-off 20 ng/mL described as most precise discriminator of health vs disease	Single/limited series + clinical use literature	Some replication across cohorts (varies by assay)	Near-clinical	Clinically attractive because it is threshold-based; still requires harmonised cut-offs across assays and populations.
**miRNA signatures (GCF/saliva)**	GCF miR-155, miR-146a; stage-linked panels	Accuracy reported for individual miRNAs (e.g., 82.6% for miR-155; 86.1% for miR-146a in diabetes context)	Mainly single studies; plus systematic reviews/meta-analytic synthesis for	Variable; limited external validation	Exploratory–Validated (marker-dependent)	Performance inflation risk in case–control designs; platform variability (RT-PCR vs arrays) and confounders.

			some candidates			
**sEV-associated miRNAs (saliva)**	3 miRNAs enriched in salivary sEVs	AUC 0.96 (periodontitis discrimination); signal not seen in whole saliva	Single pilot study	No robust external validation	Exploratory	Strong signal but early; requires replication and standardised EV isolation/pre-analytics.
**Cell-free DNA (GCF/saliva/plasma)**	cfDNA concentrations and correlations	Correlational and severity-gradient data; no pooled diagnostic AUC reported	Cross-sectional observational studies	Limited	Exploratory	Highly sensitive to pre-analytics; limited disease specificity; better suited as a panel component than stand-alone test.
**Extracellular vesicles/exosomes (host sEV cargo)**	sEV methylation (5mC), composite EV indices	"Perfect discrimination" reported for global 5mC hypermethylation in salivary sEVs; composite indices linked to severity	Single pilot study	No external validation	Exploratory	Major bottleneck is standardisation of EV isolation/characterisation; interpret as early signal, not deployable test.

Abbreviations: 5mC: 5-methylcytosine; aMMP-8: active matrix metalloproteinase-8; AUC: area under the receiver operating characteristic curve; cfDNA: cell-free DNA; EV: extracellular vesicles; GCF: gingival crevicular fluid; IL-1β: interleukin-1 beta; miR-155 / miR-146a: microRNA-155 / microRNA-146a; miRNA: microRNA; MMP-8: matrix metalloproteinase-8; ng/mL: nanograms per milliliter; POC: point-of-care; ROC: receiver operating characteristic; RT-PCR: reverse transcription polymerase chain reaction; Sens: sensitivity; sEV: small extracellular vesicles; Spec: specificity.
