# Supplementary material for: Advanced Diagnostic Technologies and Molecular Biomarkers in Periodontitis: Systemic Health Implications and Translational Perspectives
Source: J Clin Med. 2026 Feb 2;15(3):1142. doi: 10.3390/jcm15031142 (PMC12898585; doi:10.3390/jcm15031142)
Supplement: Supplementary file 1 [file jcm-15-01142-s001.zip › Supplementary Table S4.pdf]

Supplementary Table S4. Minimum reporting checklist for periodontal biomarker studies

Domain	Item	Minimum to report (practical)
**Preanalytical**	**Matrix & collection**	Sample type (saliva—stimulated/unstimulated; GCF; blood/plasma/serum; plaque), collection method, time-of-day, fasting status, restrictions (toothbrushing/smoking), clinical context (baseline vs post-treatment).
	**Site/sample definition**	For GCF/plaque: site selection (deepest sites vs index sites), number of sites pooled, dwell time, contamination handling (blood/saliva), volume estimation and normalisation strategy.
	**Processing**	Time to processing, temperature during handling, centrifugation/filtration steps, aliquoting, storage temperature (e.g., −80 °C) and storage duration, number of freeze–thaw cycles.
	**Preanalytics transparency**	Missing-data handling, below-LOQ handling, and any deviations from protocol.
**Analytical**	**Assay/platform**	Platform used (ELISA, multiplex bead array, lateral-flow/POC, MS proteomics, qPCR/NGS), manufacturer/kit where relevant, target definition (e.g., aMMP-8 vs total MMP-8), units.
	**Calibration & QC**	Calibration approach, standards and controls, batch effects, blinding (if applicable), inter-/intra-assay precision (CV), LOD/LOQ, analytical specificity (cross-reactivity).
	**EV-specific (if applicable)**	Isolation method (SEC/UC/affinity capture etc.), basic characterisation (particle and protein measures), EV marker reporting aligned with ISEV (MISEV) recommendations; how microbial OMVs were considered/controlled where relevant.
**Clinical**	**Phenotype definition / reference standard**	Clear case definitions (health/gingivitis/periodontitis), staging/grading, site-level definitions if used, reference standard for diagnosis/activity.
	**Endpoints**	Primary endpoint(s): activity/progression, response/non-response, monitoring; follow-up duration for longitudinal claims; treatment context (NSPT, SPT, adjuncts).
	**Confounders & case-mix**	Smoking, diabetes control, age, sex, medications, comorbidity profile; how confounders were controlled/adjusted; spectrum/case-mix description.
	**Validation & generalisability**	Internal vs external validation; independent cohorts; prespecified thresholds; incremental value over standard clinical assessment (staging/grading); reporting of missingness and exclusions.
	**Reporting metrics**	Discrimination (AUC), sensitivity/specificity at clinically relevant thresholds, calibration where applicable, and clinical interpretability (decision thresholds/expected use-case).

Abbreviations: AUC, area under the receiver operating characteristic curve; CV, coefficient of variation; EV, extracellular vesicle; GCF, gingival crevicular fluid; ISEV, International Society for Extracellular Vesicles; LOD, limit of detection; LOQ, limit of quantification; MS, mass spectrometry; NGS, next-generation sequencing; NSPT, non-surgical periodontal therapy; OMV, outer membrane vesicle; POC, point-of-care; QC, quality control; qPCR, quantitative polymerase chain reaction; SEC, size-exclusion chromatography; sEV, small extracellular vesicle; SPT, supportive periodontal therapy; UC, ultracentrifugation.
